# Supplementary figures and images for: Surrogate R-spondins for tissue-specific potentiation of Wnt Signaling
Source: PLoS One. 2020 Jan 8;15(1):e0226928. doi: 10.1371/journal.pone.0226928 (PMC6949110; doi:10.1371/journal.pone.0226928)

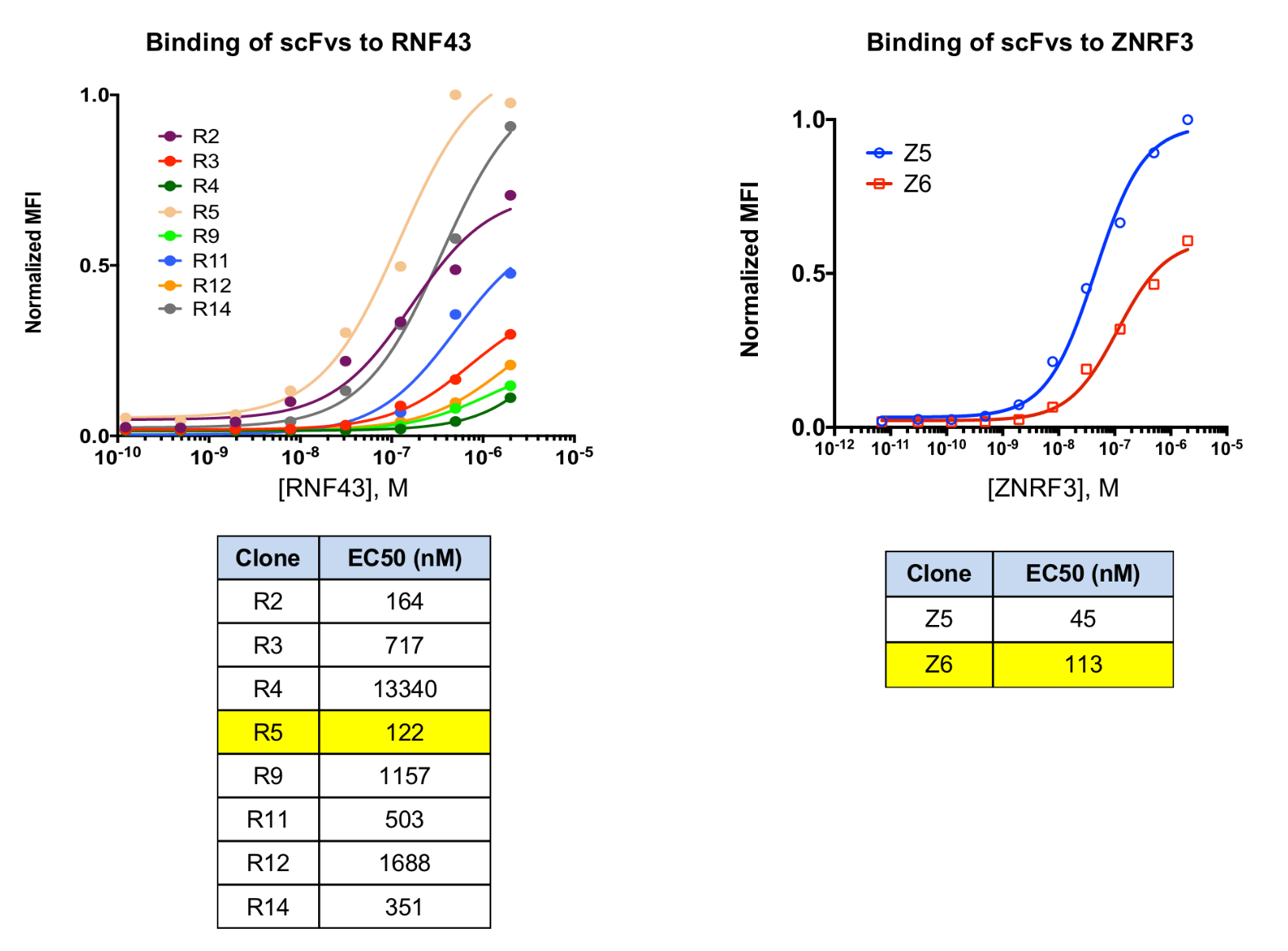

Supplement: S1 Fig — Individual clones from the yeast scFv library were titrated with increasing concentrations of RNF43 (for RNF43-selected yeast) or ZNRF3 ECDs (for ZNRF3-selected yeast) and curves were fitted to determine EC50 values (indicated in charts below). The highlighted clones were selected for incorporation into surrogate RSPOs. The Z5 clone was excluded from future studies because it encoded for a truncated scFv sequence. (PNG) [file pone.0226928.s001.png]

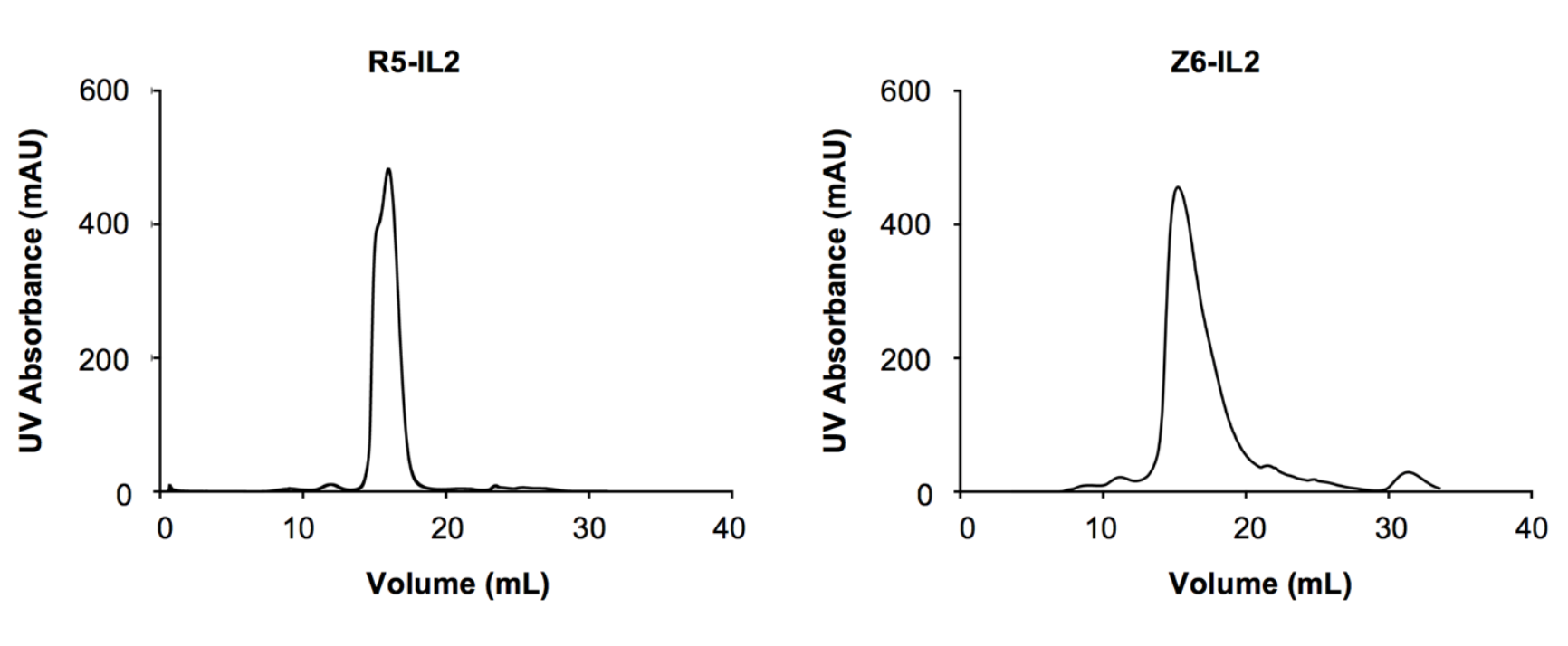

Supplement: S2 Fig — R5-IL2 (left) and Z6-IL2 (right) were injected onto Sephadex 200 gel filtration columns and UV280 absorbance was plotted versus elution volume. Both proteins eluted predominately as monodisperse peaks, which is indicative of favorable biochemical behavior. (PNG) [file pone.0226928.s002.png]

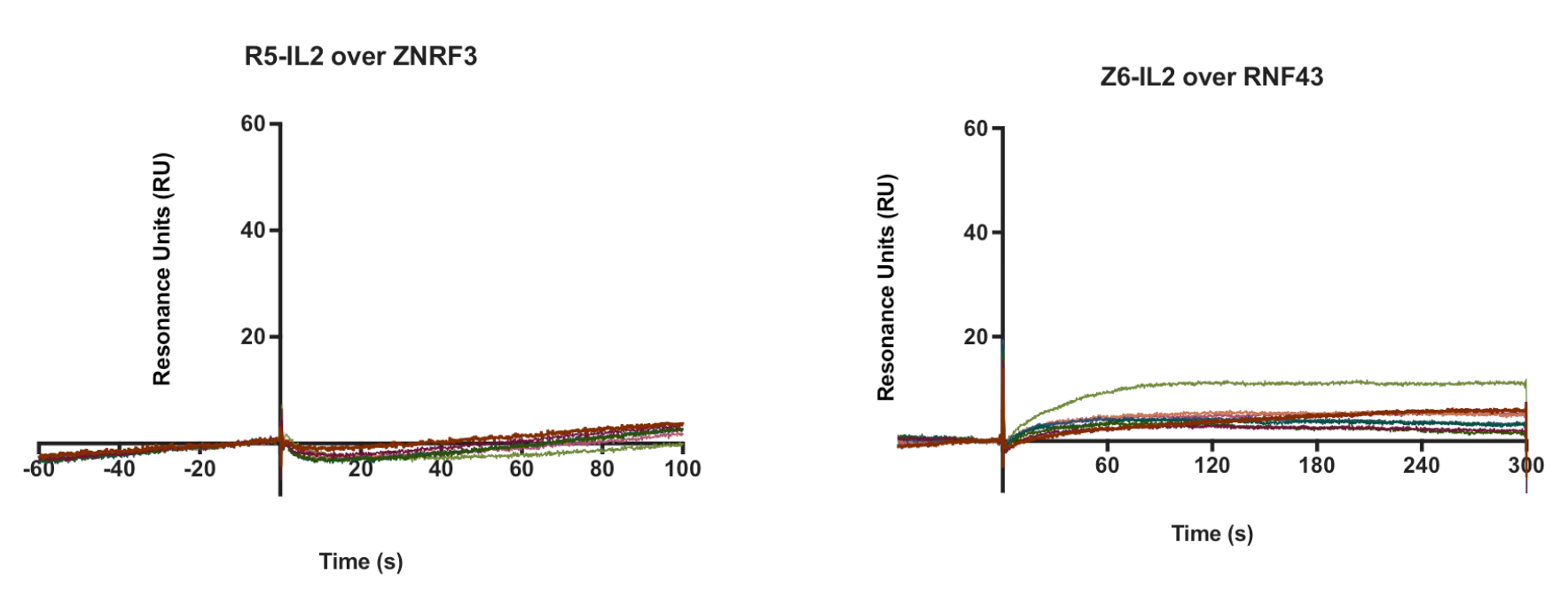

Supplement: S3 Fig — SPR was used to assess whether the R5 scFv cross-reacts with ZNRF3, and whether the Z6 scFv cross-reacts with RNF43. Increasing concentrations of R5-IL2 (3-fold dilutions, green curve is maximum concentration of 1 μM) were injected over a surface coated with the ZNRF3 ECD (left) and increasing concentrations of Z6-IL2 (2-fold dilutions, green curve is maximum concentration of 1 μM) were injected over a surface coated with the RNF43 ECD (right). Injections were performed at T = 0 seconds. R5-IL2 was flowed over the surface for 100 seconds and Z6-IL2 was flowed over the surface for 300 seconds. In both cases no substantial binding was observed, indicating that neither scFv is cross-reactive. (PNG) [file pone.0226928.s003.png]

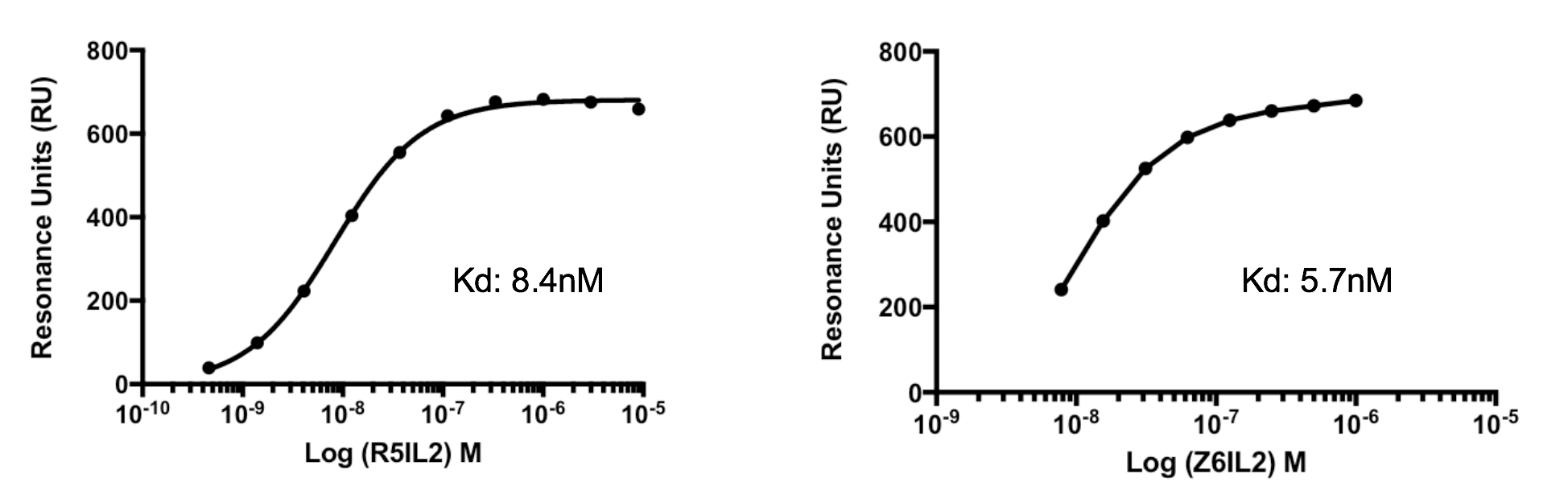

Supplement: S4 Fig — SPR was used to determine the binding affinity between R5-IL2 or Z6-IL2 and CD25. Increasing concentrations of R5-IL2 (left) or Z6-IL2 (right) were injected over a surface coated with the ECD of CD25. The maximal RU values for each curve were plotted and the binding isotherms were fitted to a 1:1 model to determine the Kd values indicated on the plots. (PNG) [file pone.0226928.s004.png]
